# Effect of daytime-restricted feeding in the daily variations of liver metabolism and blood transport of serotonin in rat

**DOI:** 10.14814/phy2.12389

**Published:** 2015-05-06

**Authors:** Marlen Valdés-Fuentes, Gabriela Vera-Rivera, Dalia De Ita-Pérez, Isabel Méndez, María Isabel Miranda, Mauricio Díaz-Muñoz

**Affiliations:** 1 Posgrado en Ciencias Biológicas, Universidad Nacional Autónoma de MéxicoQuerétaro, Qro., México; 2 Department of Behavioral and Cognitive Neurobiology, Instituto de Neurobiología, Universidad Nacional Autónoma de México (UNAM)Querétaro, Qro., México; 3 Department of Cellular and Molecular Neurobiology, Universidad Nacional Autónoma de México (UNAM)Querétaro, Qro., México

**Keywords:** Blood, daytime-restricted feeding, food entrained oscillator, tryptophan, tryptophan hydroxylase 1

## Abstract

The biogenic amine serotonin is a signaling molecule in the gastrointestinal tract, platelets, and nervous tissue. In nervous system, serotonin and its metabolites are under the control of the circadian timing system, but it is not known if daily variations of serotonin exist in the liver. To explore this possibility, we tested if the rhythmic pattern of serotonin metabolism was regulated by daytime restricted feeding (DRF) which is a protocol associated to the expression of the food entrained oscillator (FEO). The DRF involved food access for 2 h each day for 3 weeks. Control groups included food ad libitum (AL) as well as acute fasting and refeeding. Serotonin-related metabolites were measured by high pressure liquid chromatography, the anabolic and catabolic enzymes were evaluated by western blot, qPCR, and immunohistochemistry to generate 24-h profiles. The results showed in the AL group, liver serotonin, tryptophan hydroxylase-1 activity, and protein abundance as well as serotonin in plasma and serum were rhythmic and coordinated. The DRF protocol disrupted this coordinated response and damped the rhythmic profile of these parameters. We demonstrated the daily synthesis and the degradation of serotonin as well as its transport in blood. This rhythm could influence the physiological role played by serotonin in peripheral organs. DRF caused an uncoordinated response in the liver and blood serotonin rhythm. This modification could be a part of the physiology of the FEO

## Introduction

Serotonin or 5-hydroxytryptamine (5-HT) is a monoamine synthesized in the neurons of the central nervous system, as well as in the enterochromaffin cells (ECs) of the gastrointestinal (GI) tract (Mawe and Hoffman 2013). It regulates a variety of physiologic states and behaviors such as pain, appetite, mood, learning, sleep, vascular tone, GI motility and apoptosis, intestinal cell proliferation, and platelet aggregation (Berger et al. 2009; Bertrand and Bertrand 2010). 5-HT is synthesized from the essential amino acid L-tryptophan (L-Trp). The rate limiting enzyme, tryptophan hydroxylase (or L-aromatic amino acid decarboxylase, tryptophan hydroxylase [TPH] EC 1.14.16.4.) converts L-Trp to 5-hydroxytryptophan (5-HTP), which is then converted to 5-HT. TPH exists in two isoforms, TPH-1 found in the pineal gland and peripheral organs (Sakowski et al. 2006), and TPH-2 selectively expressed in brain (Walther et al. 2003). About 95% of 5-HT in the body is found in the GI tract, mostly within the ECs, and the remaining 5% is in the brain and different tissues (Lesurtel et al. 2008). Platelets are the major sites for 5-HT storage, and the 5-HT released following platelet aggregation is the source of 5-HT available for blood vessels (Ni et al. 2008). 5-HT in tissues is rapidly metabolized, mainly by monoamine oxidase (MAO) (EC 1.4.3.4.). There are two types of MAO enzymes, and MAO-A is the one that preferentially oxidizes 5-HT (Bach et al. 1988). In the liver, 5-HT regulates several key aspects of liver function, such as hepatic blood flow, innervation, wound healing, and liver regeneration (Ruddell et al. 2008; Kawasaki et al. 2010).

In mammals, circadian rhythms are generated by the major endogenous pacemaker localized in the suprachiasmatic nucleus (SCN), at the anterior hypothalamus (Challet 2007). It is now accepted that alternative clocks are located in many peripheral organs and tissues, such as the liver. The resultant timing system can be entrained by environmental factors including photonic and non-photonic stimuli (Vollmers et al. 2009). Daytime restricted feeding (DRF) is an experimental protocol used to study the physiological and metabolic adaptations of circadian rhythmicity when mealtime becomes a predominant timing cue (Stephan 2002). DRF usually involves food intake for 2–4 h in the middle of the light period. After few days, the animals show behavioral arousal before meal time known as anticipatory activity (Mistlberger 2009). In addition, the 24-h variations of a great variety of metabolic and endocrine parameters change their phase, amplitude, or even the type of rhythmicity. Overall, DRF orchestrates a physiological response that has been considered by some authors as rheostatic (Aguilar-Roblero and Díaz-Muñoz 2009). Underlying the DRF protocol is also the expression of an alternative circadian oscillator, independent of the SCN, whose anatomic location is so far unknown. This putative oscillator is known as the food entrained oscillator (FEO) (Mendoza 2007).

The aim of this study was to gain more about the daily fluctuations of serotonin metabolism in the liver when food access is ad libitum (AL) or restricted. The results showed that 5-HT is generated within the liver, and that its synthesis is regulated differently during FEO expression.

## Materials and Methods

### Animals

Adult male Wistar rats weighing 200 ± 20 g were maintained in constant conditions of 12 h:12 h light-dark cycles (lights on at 8 h) and constant temperature (22 ± 1°C). Rats were kept in groups of four in transparent acrylic cages (40 × 50 × 20 cm) with free access to water and Purina Chow except during DRF, acute fasting (Fa), or acute fasting-refeeding (Fa-Re) conditions. All experimental procedures were approved by the Bioethics Committee of the Institute of Neurobiology, Universidad Nacional Autónoma de México, and confirmed with international ethical standards (Portaluppi et al. 2010).

### Experimental design

The experimental protocol had four groups: (1) Rats fed AL; (2) rats under DRF schedule with mealtime only from 1200 to 1400 h for 3 weeks; (3) rats fasted for 21 h, and (4) rats that were fasted 22 h and refed for 2 h (from 1200 to 1400 h). Rats were sacrificed by decapitation at 0800, 1100, 1400, 1700, 2000, 2300, 0200, and 0500 h. The controls of feeding condition, Fa and Fa-Re, were sacrificed at 1100 h and 1400 h, respectively. Immediately after sacrifice, livers were homogenized or frozen in dry ice and kept at −80°C until analysis. Two samples of blood were collected. Immediately after decapitation, for serum, blood was collected in Vacutainer^®^ (Becton Dickinson, Mexico City, Mexico) tubes and centrifuged at 2500*g* for 5 min. For plasma, blood was collected in BD Vacutainer^®^ (Becton Dickinson) tubes with K_2_ Ethylenediaminetetraacetic acid (EDTA) and centrifuged at 1500*g* for 10 min.

### Subcellular fractionation

A sample of 2 g of the liver was homogenized in 15 mL of buffer (10 mmol/L Tris–HCl, pH 7.4, 225 mmol/L sucrose, 0.2% Bovine serum albumin, 0.3 mol/L Ethylene glycol tetraacetic acid). Briefly, the homogenate was centrifuged at 1500*g* for 15 min, and the supernatant was centrifuged at 10,000*g* for 15 min to sediment the mitochondrial fraction, which was resuspended. The second supernatant was centrifuged at 100,000*g* for 1 h for the microsomal and cytosolic fractions. All centrifugations were performed at 4°C, and aliquots were kept at −80°C.

### qPCR amplifications

TPH-1 gene expression was evaluated by isolating total RNA from liver tissue (20–30 mg) using the SV Total RNA Isolation System (Promega, Madison, WI). The amount and quality of RNA were estimated spectrophotometrically at 260 and 280 nm, and a constant amount of RNA (2 *μ*g) was reverse transcribed using SuperScript^™^ III Reverse Transcriptase, Oligo(dT)_12–18_ Primer, RNaseOUT^™^ recombinant ribonuclease inhibitor, and dNTP Set polymerase chain reaction (PCR) Grade (Invitrogen, Carlsbad, CA). Amplification was performed in triplicate in the CFX96TM real-time PCR detection system (Bio-Rad, Hercules, CA). Primers used for quantitative PCR (qPCR) amplifications were synthesized by Sigma-Aldrich Co. (St. Louis, MO), and the corresponding sequences are shown in Table1. Amplifications were carried out with Maxima SYBR Green qPCR Master Mix (Thermo Fisher Scientific, Waltham, MA) in a 10 *μ*L final reaction volume containing cDNA (1/20) and 0.5 *μ*mol/L of each of the primer pairs in SYBR Green Master Mix, according to the following protocol: activation of Taq DNA polymerase and DNA denaturation at 95°C for 10 min, followed by 40 amplification cycles consisting of 10 sec at 95°C, 30 sec at 60°C, and 30 sec at 72°C. The PCR data were analyzed by the 2^−ΔΔCT^ method, and cycle thresholds normalized to the housekeeping gene Rps18 were used to calculate the mRNA levels of TPH-1.

**Table 1:** *Rattus norvegicus* oligonucleotide sequences used in qRT-PCR experiments

Genes	GenBank		Primer sequence	Size (bp)	Ta (°C)
Tryptophan hydroxylase 1 (Tph1)	NM_001100634.2	Sense	GCTGAACAAACTCTACCCAAC	86	60
		Antisense	CTTCCCGATAGCCACAGTATT		
Tryptophan hydroxylase 2 (Tph2)	NM_173839.2	Sense	GGGTTACTTTCCTCCATCGGA	85	60
		Antisense	AAGCAGGTTGTCTTCGGGTC		
Monoamine oxidase A (MAO-A)	D00688	Sense	GCCAGGAACGGAAATTTGTA	231	62
		Antisense	TCTCAGGTGGAAGCTCTGGT		
Ribosomal protein S18 (Rps18)	BC126072.1	Sense	TTCAGCACATCCTGCGAGTA	136	62
		Antisense	TTGGTGAGGTCAATGTCTGC		

### Western-blot analysis

Proteins from the liver homogenate and hepatic fractions were separated by 15% sodium dodecyl sulfate polyacrylamide gel electrophoresis (SDS-PAGE) under reducing conditions, transferred to nitrocellulose membranes, and blocked for 1 h in Tris-Buffered Saline and Tween 20 (TBST) buffer (20 mmol/L Tris, pH 7.5, 500 mmol/L NaCl, 0.5% Tween 20) with 5% non-fat milk. Anti-TPH1 (ab52954, Abcam, Cambridge, U.K.) (1/3500 in TBST) or anti-MAOa (sc-20156, Santa Cruz Biotechnology, Inc, Santa Cruz, CA) (1/1000 in TBST) was added as primary antibody and incubated overnight at 4°C. As loading controls for liver homogenates, membranes were incubated with mouse anti-tubulin antibody (ab 56676 Abcam, UK) diluted 1/1000, for mitochondrial fractions was used as control protein of mitochondrial origin, rabbit anti-VDAC1/Porin antibody (ab15895; Abcam, UK) diluted 1/1000. After washing, membranes were incubated with the appropriate secondary antibodies conjugated to alkaline phosphatase (sc2315, Santa Cruz Biotechnology, Inc.)1/5000. Bands were revealed using the AP conjugate substrate kit (Bio-Rad, CA). Densitometric analysis was performed using the Image Lab Software (v 3.0, Bio-Rad, CA).

### Immunohistochemistry

Liver tissue was fixed for 1 week in 10% formalin at 4°C, with formalin changes every 2 days. After fixation, the tissue was embedded in paraffin and sectioned into 7 *μ*m slices. Samples were deparaffinized for 2 h at 60°C in a dry-heat oven. After that, sections were rehydrated in the solvent series: Xylol 100% (10 min), ethanol 100% (5 min), ethanol 96% (5 min), ethanol 80% (5 min), and deionized water (10 min), with a subsequent bath in permeabilization buffer (4 mmol/L sodium citrate, 0.1% Tween 20) for 8 min. Antigen retrieval was performed in EDTA buffer (1 mmol/L EDTA, 0.05% Tween 20, pH 8) in a water bath at 90°C for 1 h, followed by blocking with 1% non-fat milk for 1 h. The sections were washed 3 × 5 min with 0.05% TBST and then incubated overnight at 4°C with the primary antibody against TPH-1 (ab52954, Abcam, UK) diluted 1:50. The next day, sections were washed 3 × 5 min with 0.05% TBST and incubated for 2 h with the secondary antibody (Alexa Fluor^®^ 594 goat anti-rabbit IgG, Invitrogen) diluted 1:400. The staining was visualized using a confocal microscope Zeiss Axiovert 200 LSM 510 Meta-Multiphoton and captured by software LSM 510 Meta by Zeiss (Mexico city, Mexico).

### Tryptophan hydroxylase activity

TPH activity was measured in liver homogenates using a radioactive isotope assay (Barbosa et al. 2008) with the following reaction medium: Hepes (50 nmol/L, pH 7), catalase (100 *μ*g/mL), tryptophan (50 *μ*mol/L), dithiothreitol (5 mmol/L), Fe (NH_4_)_2_ (SO_4_)_2_ (10 *μ*mol/L), 6-methyl-5-6-7-8-tetrahydropterine dihydrochloride (6-MPH4) (500 *μ*mol/L), and 1 *μ*L of [^3^H] tryptophan (1 *μ*/mL). The sample was incubated at 37°C for 10 min. The reaction was stopped by adding a suspension of activated charcoal (7.5% in 1 mol/L HCl), and 200 *μ*L of the supernatant was transferred to scintillation tubes; scintillation fluid was added, and radioactivity was evaluated with a Beckman *β* counter.

### Measurement of 5-HT, 5-HTP, and L-Trp by HPLC

5 HT and 5-HTP concentrations within the liver, platelet-free plasma, and platelet-rich plasma were assayed by high pressure liquid chromatography (HPLC). Liver samples were homogenized 1/5 (w/v) in 0.1 mol/L HClO_4_ containing 0.2 mmol/L EDTA, filtered with a centrifuge tube filter (Spin-X, Costar^®^, Corning, NY). The HPLC system consisted of a delivery pump (Solvent Delivery System PM-80; Bioanalytical Systems, West Lafayette, IN), a simple injector (BASi Liquid Chromatography CC-SE, 20 *μ*L loop; Bioanalytical Systems), a C18 reverse phase column (BASi ODS C18, 100 × 3 mm, 3 *μ*m particle size; Bioanalytical Systems), and an electrochemical detector (Bioanalytical Systems) with a carbon electrode; the potential was adjusted to +600 mV versus the reference electrode (Ag/AgCl). The mobile phase contained 0.1 mol/L Na_2_HPO_4_, 0.05 mol/L Citric acid, 0.17 mmol/L EDTA, 1 mmol/L KCl, and 2% methanol (v/v), pH 4.5. The flow rate was 0.6 mL per min at a pressure of 2400 psi. All chromatograms were recorded and analyzed using the ChromGraph Report software 2.3 (Bioanalytical Systems). Tryptophan was measured in homogenates using HPLC (Dionex, Ultimate 3000, Thermo Scientific, Chromeleon 6.8 software integrator for data analysis, Waltham, MA) with fluorescence detection (excitation and emission 337 and 442 nm, respectively) after o-phthaldehyde (OPA) derivatization. The sample homogenates (25 *μ*L) were mixed with 25 *μ*L of OPA in a tube, and after 2 min they were injected onto a Syncronis C18 column (250 × 4.6 mm, Particle size 5 *μ*m, Thermo Scientific) at 20°C. We used a mobile phase of 0.1 mol/L potassium acetate adjusted to pH 5.5 with glacial acetic acid and a gradient with 25% methanol, 75% mobile phase, flow rate 2.5 mL/min.

The concentrations were determined from peak areas using external standards of L-Trp, 5-HTP, and, 5-HT (Sigma-Aldrich Co., St. Louis, MO).

The protein concentrations are expressed as nmol/*μ*g protein and pmol/*μ*g protein in liver homogenates and nmol/mL for blood determinations.

### Platelet count

Platelets in the blood samples were quantified by a standard clinical procedure using an automated cell counter.

### Statistical analysis

Results are expressed as the mean ± SEM. The statistical analyses were performed using GraphPad Prism software (v 5.0; San Diego, CA). Normality distribution and equal variances were determined by the Kolmogorov–Smirnov and Levene tests. Different time points were compared using one-way analysis of variance (ANOVA), and groups were compared using a two-way ANOVA test followed in both cases by a post hoc Bonferroni test. All pairwise multiple comparisons were performed by the student's *t*-test. Differences among groups were considered statistically significant at *P* ≤ 0.05. Rhythm analyses were performed by COSANA software (v 3.1 developed by AA Benedito-Silva, GMDRB, ICB/USP, Sao Paulo, Brazil).

## Results

### Daily variations of liver L-Trp, 5-HTP, and 5-HT in rats under daytime-restricted feeding

**Figure 1:**
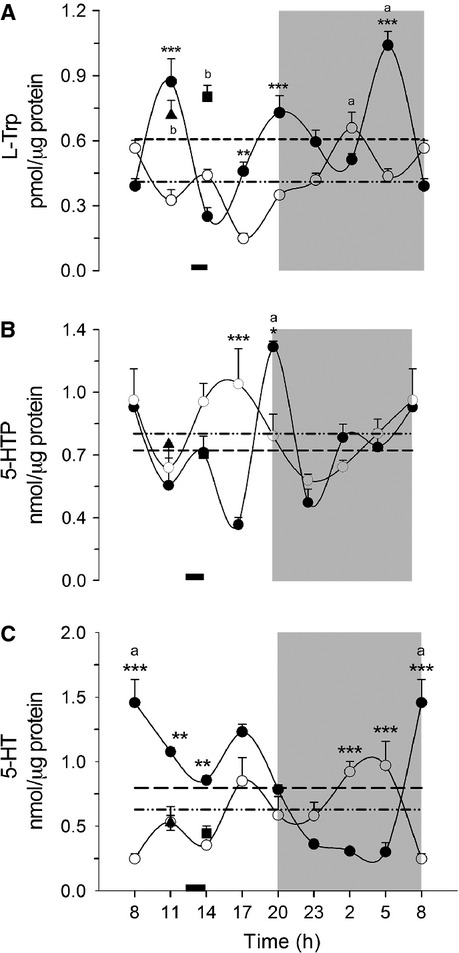
Analysis of the daily variation of L-Trp (A), 5-HTP (B) and 5-HT (C) in rat liver measured by HPLC. Each point represents the mean ± SEM. Access to food for the DRF group is represented by the black bar (1200–1400 h). AL (●), DRF (○), Fa (▲), Fa-Re (■), mesor AL(─) DRF (−∙∙), (a) one-way ANOVA, post hoc Bonferroni test. *P < 0.05, **P < 0.01, ***P < 0.001, two-way ANOVA, post hoc Bonferroni test. (b) Student's *t*-test *P* < 0.05. *n* = 4. 5-HTP, 5-hydroxytryptophan; 5-HT, 5-hydroxytryptamine; HPLC, high pressure liquid chromatography; DRF, daytime restricted feeding; ANOVA, analysis of variance.

The precursor for serotonin is L-Trp. In Figure1A, it can be seen that hepatic L-Trp in the AL group showed a pattern of three peaks (1100, 2000 and 0500 h) with valleys at 1400 and 0800 h. DRF promoted a drastic change in the rhythmic pattern, showing a clear valley at 1700 h and higher levels of L-Trp during the last part of the dark period. There were significant differences between the two groups at 1100, 1700, 2000, and 0500 h, corresponding to the points of highest concentration in the AL group (two-way ANOVA *P* < 0.05, *F*_(7, 42)_ = 18.33). Another difference associated with the DRF protocol was a significant reduction (~50%) in the average levels of L-Trp in the 24-h period. The cosinor program indicated a 47% adjustment in the AL group with a period of 8 h, whereas the DRF group showed an adjustment of 56% with a period of 24 h (Table2). Control groups of feeding condition (Fa and Fa-Re) showed L-Trp levels more similar to those of AL rats, that is, significantly higher than the corresponding levels in DRF rats, both before food access (~115% at 1100 h) and after feeding (~82% at 1400 h) (Student's *t*-test *P* = 0079 and 0.0096; *P* < 0.05).

**Table 2:** Chronobiological analysis of liver and blood samples

	L-Trp liver		5-HTP liver		5-HT liver		Enzymatic activity TPH		MAO-A mRNA		5-HT serum		5-HT plasma		Count of platelets	
	AL	DRF	AL	DRF	AL	DRF	AL	DRF	AL	DRF	AL	DRF	AL	DRF	AL	DRF
Period	8	24	12	12	24	12	24	24	24	24	24	24	24	24	8	24
Amplitude	0.24	0.14	0.19	0.19	0.45	0.21	12	8.87	1.35	0.65	97	92	6	5	317	197
Mesor	0.60	0.41	0.73	0.82	0.80	0.63	22	17.5	4.2	1.3	19	176	13	13.5	1269	1404
% rhythm	47.2	55.7	21	50	58	35	50	75	43	54	85	75	60	44	66	48

L-TRP, L-tryptophan; 5-HTP, 5-hydroxytryptophan; 5-HT, serotonin; TPH, tryptophan hydroxylase; MAO-A, monoamine oxidase isoform A; DRF, daytime restricted feeding.

5-HTP is an intermediate in the conversion of L-Trp to 5-HT. Figure1B displays the daily variations of liver 5-HTP. The group AL showed a pattern with two clear peaks, at 0800 and 2000. The peak at 0800, at the beginning of the light period, is reached by gradual changes that occur over 1800 h (from 2300 to 1700 h); in contrast, the peak at 2000 h, at the beginning of the dark period, is sudden and limited to a single time point. The DRF protocol changed some aspects of the rhythmic pattern of 5-HTP: it also showed two peaks, one at the beginning of the light period (0800 h) and similar to the one shown by AL rats; however, the second peak was observed at 1700 h, different from the AL group. Another difference was that in the case of the DRF rats, the two peaks were shallower, since both showed incremental changes in the 5-HTP levels. As a consequence, the pattern in the AL rats was better adjusted, by the cosinor program, to a 12-h period but with low rhythmicity (~21%), whereas the temporal variations of 5-HT in DRF rats was also adjusted to a 12-h period but with higher rhythmicity (~50%) (Table2). Significant differences between the two treatments were detected at 1700 and 2000 h, with no significant change in the 24-h average value in either group (two-way ANOVA *P* < 0.05; *F*_(7, 42)_ = 7.232). 5-HT levels were sensitive neither to Fa nor to the subsequent refeeding condition.

Daily variations of liver 5-HT in DRF rats are shown in Figure1C. The AL group showed a well-defined cycle with minimum 5-HT concentrations during the dark period, and significantly higher levels during the light period (one-way ANOVA *P* < 0.05, *F*_(7, 24)_ = 33.97, post hoc Bonferroni's test). A clear peak was observed at 0800 h, during the transition from dark to light periods. This pattern was drastically altered by the DRF protocol, since concentrations of 5-HT were lower in the light period and higher during the dark period. Significant differences between the two treatments occurred at 0800, 1100, 1400, 0200, and 0500 h (two-way ANOVA *P* < 0.05: *F*_(7, 24)_ = 18; post hoc Bonferroni ‘*s* test). The 24-h rhythm of the AL group was better fitted to a sinusoidal pattern (~50% with a 24-h period) than the rhythm shown by the DRF group (~36% with a 12-h period) (Table2). A non-significant tendency for the 24-h-average value to be reduced in the DRF group was observed. Fa and Fa-Re groups showed values more similar to the DRF group.

One important aspect of this analysis is that the concentration of the precursor amino acid was lower than the concentrations of 5-HTP and 5-HT, suggesting that this amino acid is rate-limiting the 5-HT metabolism.

### Daily variations of liver TPH in rats under DRF

**Figure 2:**
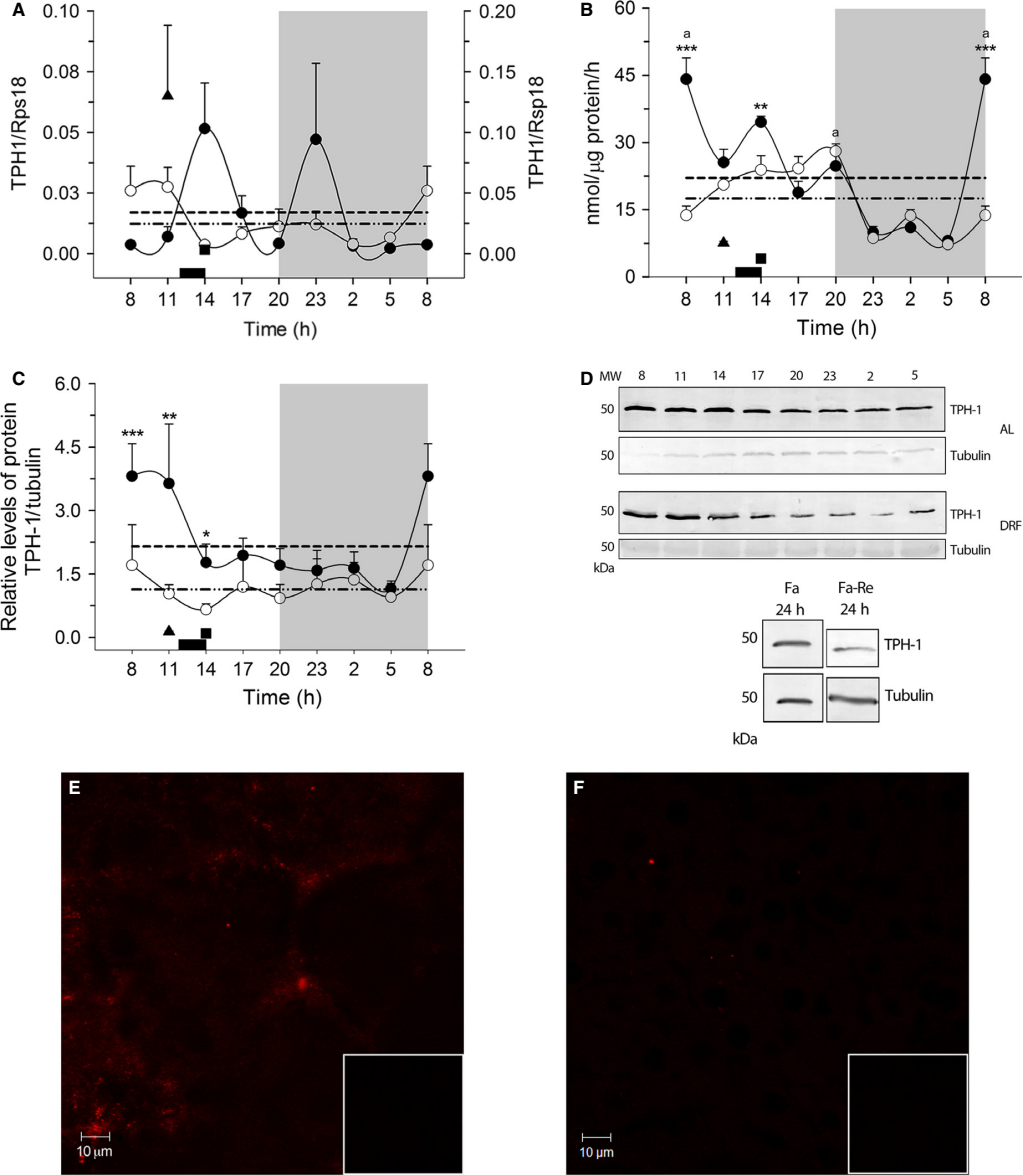
Daily variation in TPH mRNA expression (A) scale at right for Fa value, enzymatic activity (B) protein (C and D), and immunohistochemistry (E and F) in liver. Each point represents the mean ± SEM. Access to food for the DRF group is represented by the black bar (1200–1400 h). AL (●), DRF (○), Fa (▲), Fa-Re (■), mesor AL(─) DRF (−∙∙), (a) one-way ANOVA, post hoc Bonferroni test and Kruskall–Wallis test. *P < 0.05, **P < 0.01, ***P < 0.001, two-sample Kolgomorov–Smirnov test. (b) Mann–Whitney *P* < 0.05. *n* = 4. In E and F, the box at the lower right shows the negative control. TPH, tryptophan hydroxylase; DRF, daytime restricted feeding; ANOVA, analysis of variance.

Figure2 depicts the daily profile of biochemical parameters of TPH-1, the rate-limiting enzyme for serotonin synthesis. Figure2A shows the 24-h variation of the hepatic TPH-1 mRNA. The AL group showed a bimodal pattern with peaks during the light period (at 1400 h) and during the dark period (at 2300 h). The DRF protocol promoted a lack of rhythmicity in the profile for TPH-1 mRNA. It was noteworthy that Fa increased the TPH-1 mRNA significantly; this effect was reversed by the 2-h food access. TPH-2 mRNA did not show amplification in liver samples (data not shown).

Figure2B shows the daily variations of TPH activity in liver homogenates, as measured by a coupled enzymatic assay. The 24-h profile in the AL group indicated a clear rhythm with a peak at the transition between dark and light periods (0800 h). There was a significant drop during the dark period (one-way ANOVA *P* < 0.05, *F*_(7, 22)_ = 30.69, Bonferroni ‘s post hoc test). The profile of the DRF group was similar, with one notable exception: the 0800 h peak was not observed; thus, TPH activity was significantly lower in the rats under DRF. Another significant reduction was detected at 1400 h (two-sample Kolgomorov–Smirnov test, *P* < 0.05). In general, the DRF group also had higher values during the light period (Kruskal–Wallis test = 25.11, *P* = 0.0007, *P* < 0.05). Both groups were adjusted to a 24-h rhythm, with a rhythmicity of 50% for the AL group and 75% for the DRF group (Table2). Control groups of feeding conditions showed significantly lower values than the AL and DRF groups.

The daily variations of the TPH-1 protein were evaluated in the liver cytosolic fraction by western blot assay (Fig.2C). Anti-TPH-1 antibody detected a defined band with the expecting molecular weight (~50 kDa). Similar to the TPH activity, AL rats showed a well-defined rhythm for the amount of liver TPH-1 protein, with an elevation from 0800 to 1100 h, and significantly lower levels at other times (from 1400 to 0500 h). DRF rats did not show a rhythmic pattern for cytosolic TPH-1, and it was present at considerably lower levels than in the AL group during the first part of the light phase (from 0800 to 1400 h) (two-sample Kolgomorov–Smirnov test, *P* < 0.05). The TPH-1 signal in the acute Fa and Fa-Re rats showed levels similar to those for the DRF group, and significantly lower than those for the AL rats. Immunohistochemical staining for the TPH-1 protein in liver showed lower intensity in the DRF than in the AL condition, confirming the results observed in Western-blot assay (WB) and enzymatic activity assays (Fig.2E and F).

### Daily variations of MAO-A in rats under DRF

**Figure 3:**
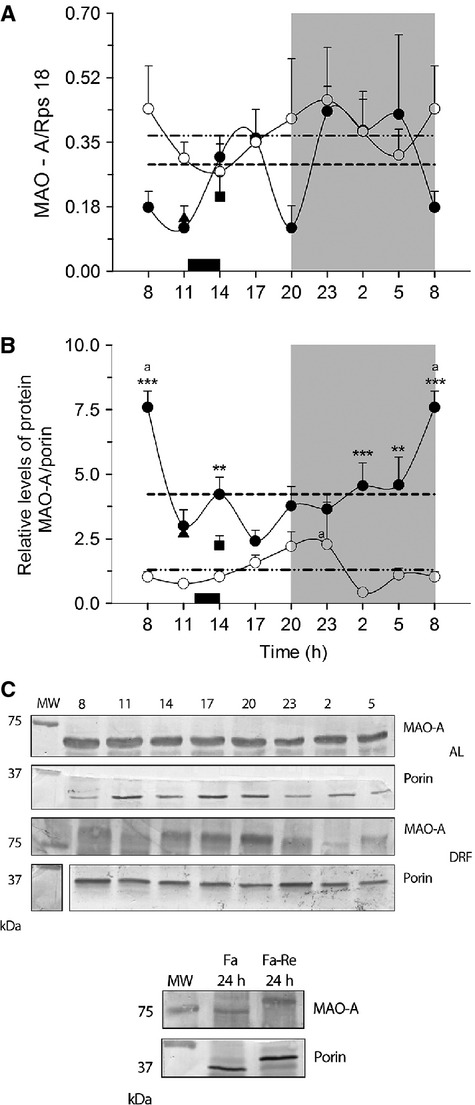
MAO-A in the rat liver. (A) Levels of MAO-A mRNA expression and (B) protein in mitochondria (C) Blot representative sample of representative abundance of MAO-A protein in the different conditions. Each point represents the mean ± SEM. Access to food for the DRF group is represented by the black bar (1200–1400 h) AL (●), DRF (○), Fa (▲), Fa-Re (■), mesor AL(─) DRF (−∙∙), (a) Kruskall–Wallis test. *P < 0.05, **P < 0.01, ***P < 0.001, Kolgomorov–Smirnov two-samples test. (b) Mann–Whitney *P* < 0.05. *n* = 4. MAO, monoamine oxidase; DRF, daytime restricted feeding.

Figure3 shows the 24-h mRNA and protein profiles of the main catabolizing enzyme for serotonin in the liver, mitochondrial MAO-A. The AL rats showed a bimodal rhythm in the expression of MAO-A mRNA (Fig.3A). Two peaks were detected, one during the light period (at 1400–1700 h) and the second throughout the entire dark period (from 2300 to 0500 h). In contrast, in the DRF group the rhythmicity was lost. No significant differences were detected. In the same way, the mRNA for MAO-A in the control groups of feeding conditions did not show any change, and were similar to the AL and DRF groups.

The daily profile of MAO-A protein assayed by western blot of the liver mitochondrial fraction is shown in Figure3B. The AL group showed a well-defined rhythm with a peak at 0800 h and a plateau with lower values from 1100 to 0500 h. The pattern in the DRF rats was very different: the relative abundance of MAO-A protein was significantly lower over the entire 24-h period, and a single peak at the beginning of the dark period was detected (2000–2300 h). Both groups were significantly different at 0800, 1400, 0200, and 0500 h (Kolgomorov–Smirnov two-samples *P* < 0.05). Cosinor analysis indicated similar rhythmicity in both groups: 43% in AL rats and 54% in DRF rats. Acute Fa and Fa-Re showed similar levels of MAO-A protein, which were intermediate between the AL and the DRF group (Mann–Whitney test *P* = 0.0379).

### Circulating 5-HT: differences between plasma and serum measurements

**Figure 4:**
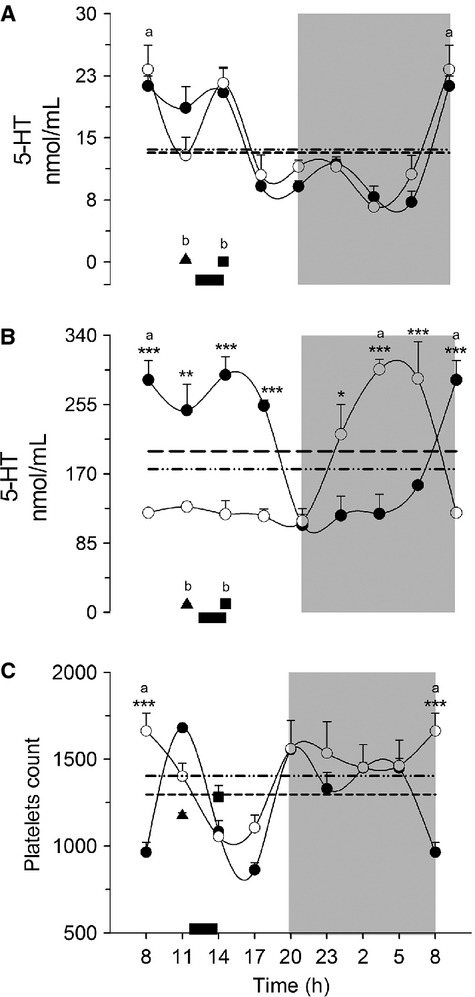
Concentration of 5-HT in blood. Plasma (A), serum (B), and number of platelets (C). Each point represents the mean ± SEM. Access to food for the DRF group is represented by the black bar (1200–1400 h) AL (●), DRF (○), Fa (▲), Fa-Re (■), mesor AL(─) DRF (−∙∙), (a) one-way ANOVA, post hoc Bonferroni test. *P < 0.05, **P < 0.01, ***P < 0.001, two-way ANOVA, post hoc Bonferroni test. (b) Student's *t*-test *P* < 0.05. *n* = 4. 5-HT, 5-hydroxytryptamine; DRF, daytime restricted feeding; ANOVA, analysis of variance.

Blood samples analyzed for plasma were pre-treated with EDTA. As shown in Figure4A, the levels of plasma 5-HT were very similar in the AL and DRF rats. Both showed a marked rhythm with higher 5-HT during the light period (one-way ANOVA *P* < 0.0001, *F*_(7, 23)_ = 11.66 and *P* < 0.0001, *F*_(7, 22)_ = 8.072, post hoc Bonferroni ‘*s* test) and a rhythmicity of 60% in AL group and 44% in the DRF group (Table2). In contrast, Fa and Fa-Re showed notably lower values of 5-HT (student's *t*-test *P* = 0.028). This profile was strikingly different in serum (Fig.4B). In the AL group, serum and plasma levels of 5-HT showed similar patterns; in contrast, the DRF protocol promoted an inverse profile with higher values during the dark period and lower values during the light period (one-way ANOVA *P* < 0.05, *F*_(7, 24)_ = 14.56 and *F*_(2, 22)_ = 11.07, Bonferroni's post hoc test). Both groups were significantly different at seven time points (two-way ANOVA *P* < 0.05, *F*_(7, 24)_ = 21.26, Bonferroni's post hoc test). The concentration of 5-HT in serum was 10 times higher than in plasma. Both groups had high rhythmicity based on the cosinor analysis: 85% and 75% for AL and DRF groups, respectively. In the plasma fraction, a marked reduction in the 5-HT values in the control groups of feeding conditions (student's *t*-test *P* = 0.028) was also observed.

To determine if the difference between the 5-HT plasma and serum profiles was associated with changes in the platelet count, the daily profile of these cells was evaluated (Fig.4C). Except for one time point (0800 h, with higher values of 5-HT in the DRF rats), the profile of both treatments was very similar (one-way ANOVA *P* < 0.05, *F*_(7, 20)_ = 14.60 and *F*_(7, 20)_ = 4.065, post hoc Bonferroni test). The AL group showed a cycle with 66% of rhythmicity (8-h period), whereas the DRF group had ~48% rhythmicity (24-h period) (Table2). No differences were observed with the control groups of feeding conditions.

## Discussion

This study provides the first data concerning the daily variations in the presence and activity of key elements of serotonin-related metabolites and serotonin-metabolizing enzymes in the rat liver. Most of the parameters studied showed clear fluctuations over the 24-h period in AL conditions. By other hand, these rhythms were significantly damped under the DRF protocol. Besides the rhythmic profile within the liver, 5-HT also showed daily fluctuations in its blood transportation. This parameter was deeply modified by the DRF protocol involving important adaptations in the platelets capacity to handle serotonin.

### Restricted food intake reduced the concentration of liver tryptophan and serotonin

The liver gives feeding cues, participates in digestive process, and is a peripheral integrator of the nutrient availability of the organism (Langhans 1996). L-Trp needs to be supplied within the diet (Cansev and Wurtman 2007). L-Trp is also a crossroad metabolite: in the liver, it can be incorporated into proteins, be transformed in the kynurenine pathway, responsible for almost 90% of tryptophan catabolism (Sainio et al. 1996), and act as the precursor for the synthesis of 5-HT. More recently, it was reported that L-Trp acts as precursor of 5-methoxyindole metabolites that control cyclooxygenase expression, inflammation, and tumorogenesis (Wu et al. 2014). Figure1 shows that the hepatic levels of L-Trp were ~1000-fold lower than those of 5-HTP and 5-HT. Low levels of liver L-Trp are suggestive that the compartmentalization and availability of this amino acid could be a regulatory factor, in addition to the activity of its metabolizing enzymes. At some time points, DRF promoted a marked reduction in L-Trp and 5-HT that was not observed in 5-HTP.

Physiological roles of 5-HTP in the brain have not been reported, but in the gut, it contributes to the development of intestinal microvilli (Nakamura et al. 2008). In addition, some antioxidant properties have been associated with 5-HTP. It is evident that DRF induces a new steady state in the liver L-Trp – 5-HT system as shown by the cellular uptake and exit events, as well as the enzymatic transformation within the hepatocytes. The new metabolic status of L-Trp and 5-HT in the liver under the DRF protocol can also be deduced from the different response that these metabolites showed in the control groups of feeding conditions: whereas the L-Trp levels after the Fa and Fa-Re were similar to the AL rats, the levels of 5-HT in these two groups resembled those of the rats under DRF (Fig.1).

### DRF is associated with reduced protein and activity of liver TPH-1

In normal circumstances, the concentration of L-Trp is considerably lower than the *K*_*m*_ of TPH; hence, the enzyme is never saturated with the substrate (Tyce 1990). The isoform present in the liver is exclusively type 1; TPH-2 was not found by PCR experiments (Fig.2A, Table1). Although TPH-1 can be regulated by phosphorylation (Huang et al. 2008), our results showed parallelism between the amount of the protein and its activity in AL and DRF groups (Fig.2B and C). Thus, the data suggest that the daily variation of TPH-1 activity in the AL group and the reduced activity observed in the first half of the light period in the DRF group reflect the amount of TPH-1 protein. However, the levels of TPH-1 do not seem to correlate with transcriptional activity since the corresponding mRNA did not show these same patterns (Fig.2A). Therefore, the variations of TPH-1 must be related to post-transcriptional regulatory events, such as changes of the enzyme stability or degradation (Huang et al. 2008).

### Circulating serotonin: role played by the platelets

A remarkable finding of this study was the change in the daily levels of blood 5-HT promoted by the DRF protocol (Fig.4A and B). Whereas the daily pattern of free 5-HT in plasma samples (outside of platelets) in AL and DRF groups was similar (Fig.4A), an anti-phase rhythm was observed in the DRF group when the 5-HT content within the platelets was quantified (Fig.4B). Indeed, the levels of 5-HT in serum were much higher than those measured in plasma. The daily pattern of serum 5-HT in our AL group was similar to that reported by Sanchez et al. (2008). Since the amount of platelets was similar in control and experimental conditions (Fig.4C), the mechanism underlying the modification in 5-HT rhythmicity in the DRF group must involve a profound change in 5-HT uptake and release by the platelets. Regarding this point, it has been reported that 5-HT transporter in the platelet's plasma membrane is the principal control point from 5-HT uptake from the blood plasma (Mercado and Kilic 2010). This point is not further addressed in this study, but it is highly probable that molecular events related to the transfer of 5-HT from ECs to platelets, as well as the systemic release of 5-HT from the platelets to other tissues is influenced by the metabolic and physiological adaptations associated with the DRF protocol/expression of the FEO.

### 5-HT is synthesized within the liver

The presence of TPH-1 mRNA and the corresponding protein and enzymatic activity indicate that hepatic tissue has the capacity to generate 5-HT locally. The expression of TPH-1 mRNA in fasting liver was higher than in the group with food restriction. This fact was not reflected in the amount of the protein, suggesting changes in the mRNA translation or in protein stability. It has been reported that in brain, phosphorylation of TPH-1 can increase its stability (Nexon et al. 2011). Data from our laboratory show a coincidence between the temporal pattern of circulating glucocorticoids and TPH-1 expression during the DRF protocol, suggesting a potential control of THP-1 synthesis in the liver by corticosterone (Luna-Moreno et al. 2012). The lower value of TPH activity in the DRF group also coincided with a lower amount of the enzyme protein, suggesting that the effect associated with the DRF protocol is mainly to stabilize the TPH-1 protein. Another important aspect for the enzymatic activity is the substrate. The concentration of L-Trp depends on the ingested food, which is 30% lower in the DRF group than in the AL animals. TPH activity also depends on BH4, which acts as co-substrate; however, the mechanism for maintaining the cytosolic level of BH4 is not well understood (Nakamura and Hasegawa 2009).

In the liver, the expression of MAO-A mRNA in the DRF group is higher than in the AL group (Fig.3A). Some evidence has shown that steroid and non-steroid hormones are involved in up-regulate MAO gene expression, such as androgen and glucocorticoid in neuronal cell models (Shih et al. 2011); hence, DRF could promote an elevation of MAO-A transcription by increasing the glucocorticoid-associated signaling (Shih et al. 2011; Luna-Moreno et al. 2012). Interestingly, the DRF protocol significantly reduced the amount of mitochondrial monoamine oxidase isoform A (MOA-A) protein, as detected in western-blot experiments. This result again indicates a differential effect of DRF/FEO expression on the transcriptional activity and the stability of the mature protein.

Therefore, our results indicate that the liver is able to transform L-Trp to 5-HT, an action that is under circadian regulation. Besides, this rhythmic pattern is influenced by the DRF protocol and potentially by the FEO expression. It has been reported that hepatic tissue also has the ability to synthetize melatonin (Sanchez-Hidalgo et al. 2009), a signaling factor derived from 5-HT.

**Figure 5:**
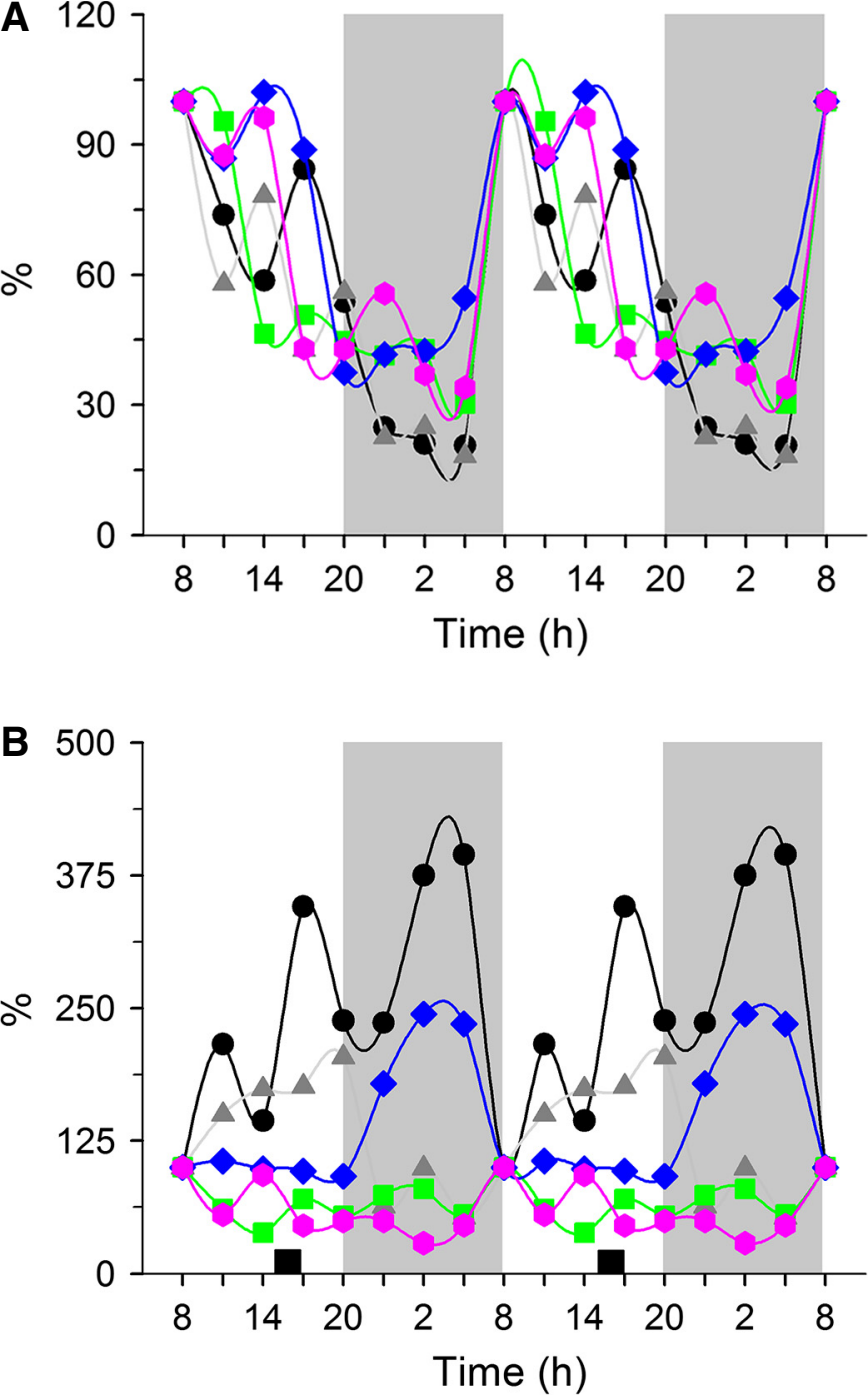
Parameters of serotonin metabolism and transportation. (A) AL and (B) DRF group. Each point represents the average percentage of the value at the first time point, for each parameter analyzed. (●) 5-HT in liver, (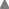) Enzymatic activity of TPH-1, (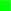) Relative level of TPH-1 protein, as well as (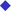) 5-HT in serum and (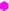) 5-HT in plasma. DRF, daytime restricted feeding; 5-HT, 5-hydroxytryptamine; TPH, tryptophan hydroxylase.

To make evident the coordinated rhythms of 5-HT in liver, serum and plasma as well as the levels and activity of TPH-1, a graphic was made with all the points normalized at values corresponding at 0800 h (the peaks or acrophases of each parameter) (Fig.5). The high synchronization of the five temporal profiles with a peak at 0800 h and higher values during the light period. Remarkably, the coordination of these 24 h-rhythms is lost by the DRF protocol. It remains to be determined if this disruption is or not associated to the expression of the FEO as well as to a possible modification in the liver physiology.

### 5-HT in the liver and food circadian synchronization

Organisms have developed mechanisms for survival under the adverse conditions of changing environments; these adaptations are manifested via circadian fluctuations (~24-h) in the concentration and activity of a number of metabolites and signaling molecules (Kirsz and Zieba 2012). It is well-documented that the liver and other peripheral organs act as circadian oscillators in coordination with the master circadian pacemaker, the hypothalamic SCN (Damiola et al. 2000). Synchronization of the peripheral circadian oscillators is also sensitive to nonphotic stimuli such as changes in food availability and temperature (Yoo et al. 2004). The protocol used in this study, feeding restricted to daytime hours, is widely accepted to promote the expression of the FEO (Mistlberger 1994, 2009; Damiola et al. 2000). Liver is one of the organs whose metabolic performance is modified more rapidly in response to the DRF protocol/FEO expression (Diaz-Muñoz et al. 2000; Baez-Ruiz et al. 2005; Vollmers et al. 2009).

Previous reports had shown that 5-HT and melatonin had clear 24-h rhythmicity in the pineal gland (Huang et al. 2008). However, this study is the first to explore the daily rhythm of 5-HT, its related metabolites and metabolizing enzymes, as well as its blood transportation (within and outside of platelets). In addition, it was clearly shown that the DRF protocol and the concomitant FEO expression affected the rhythmic variation of these parameters. From the perspective of biological rhythms, our data make two main contributions:

1. A coordinated variation of several parameters related to 5-HT metabolism and transportation was detected in AL conditions. Most of them were highly rhythmic over a 24-h period (Table2). Indeed, low levels of liver metabolites, enzymatic activities, and circulating 5-HT were observed during the dark period followed by an evident peak at 0800 h, at the beginning of the light period (Fig.5A).
2. The coordinated pattern of 5-HT-related parameters was disrupted by the DRF (Fig.5B), suggesting a possible adaptation by the liver metabolism and blood transportation of 5-HT promoted by FEO expression. As the peak at 0800 h observed in the AL group was absent, most of the parameters studied showed lower values in the DRF group, especially during the light period.

Our data show that 5-HT is generated within the liver, and its production is controlled over the 24-h period. This data could have implications in the capacity of hepatic tissue to be regenerated since 5-HT has been shown to facilitate the recovery of the hepatic tissue after surgical hepatectomy (Papadimas et al. 2006). In addition, the DRF condition significantly modifies the way in which 5-HT is produced in the liver as well as how it is transported within the circulating platelets. It is very probable that these adaptations in 5-HT metabolism and handling could be associated with the physiology of the FEO, but additional experiments are needed to support this notion. In a more general perspective, the daily control of 5-HT synthesis in the liver and blood transport opens the possibility of temporal regulation of 5-HT in other peripheral functions such as gut motility and immune modulation, bone differentiation/proliferation, and mammary gland and pancreas cell homeostasis (Amireault et al. 2013).
